# Does Procrastination Always Predict Lower Life Satisfaction? A Study on the Moderation Effect of Self-Regulation in China and the United Kingdom

**DOI:** 10.3389/fpsyg.2021.690838

**Published:** 2021-07-06

**Authors:** Zeyang Yang

**Affiliations:** ^1^Department of Psychology, School of Education, Soochow University, Suzhou, China; ^2^Department of Education, University of York, York, United Kingdom

**Keywords:** procrastination, self-regulation, wellbeing, moderation, cultural difference

## Abstract

**Aims:** Studies have shown the predictive effects of procrastination and self-regulation on wellbeing. However, little is known about the interactive effect between procrastination and self-regulation. This study explores whether self-regulation moderates the link between procrastination and wellbeing among British and Chinese young adults.

**Methods:** This study adopted self-reported questionnaire survey among two hundred and sixty-five British and four hundred and seventy-five Chinese participants. SPSS and AMOS were used to test the moderation effect. Multi-group path analysis was used to compare the two countries.

**Results:** Data analysis shows that self-regulation was a significant moderator of the relationship between procrastination and life satisfaction in the Chinese sample but not in the British sample. Procrastination predicted low life satisfaction only among the Chinese students with low self-regulation.

**Discussion:** This study indicates that the effects of procrastination on wellbeing could be changed at different levels of self-regulation. Cultural difference can be an important factor when investigating procrastination and its impacts.

## Introduction

Procrastination has been a widely explored topic among young adults in universities. It seems to be a problem of this generation as over 70% of students procrastinate (Steel and Ferrari, 2013; Habelrih and Hicks, 2015). The theoretical model of academic procrastination developed by Schraw et al. (2007) indicates the potential positive or negative effects of procrastination on people’s life quality. For example, students who procrastinate can be more engaged and efficient in their tasks when deadlines approach, and they would gain more satisfaction after finishing the tasks (Czikszentmihalyi, 1990; Brinthaupt and Shin, 2001; Chu and Choi, 2005; Abramowski, 2018). On the other hand, negative effects of procrastination are also prevalent. For example, procrastination has been shown to predict lower levels of life satisfaction (Balkis, 2013), lower wellbeing, and higher stress (Sirois and Tosti, 2012; Duru and Balkis, 2017; Çelik and Odaci, 2020).

Self-regulation, as a close companion of procrastination (Sirois and Pychyl, 2013), has also been proved to affect people’s wellbeing (De Ridder and Gillebaart, 2017). Self-regulation also appears to be a key factor to ameliorate the negative effect of procrastination. Students with sufficient self-regulation skills reported higher sense of achievement and satisfaction at the end of the term because they were proud of their finished work in a limited period (Schraw et al., 2007). In other words, without strong self-regulation, those students cannot finish their tasks in a short time. Thus, it seems necessary to investigate the interaction effect of self-regulation and procrastination on wellbeing because self-characteristics (e.g., self-regulation) can be the antecedents for procrastination and change its mechanism (Schraw et al., 2007). Furthermore, though difference was found for procrastination among East and West countries (e.g., Klassen et al., 2009, 2010), the effects of procrastination remain unknown when its interaction with self-regulation is considered. Thus, it seems important to investigate whether cultural difference exists in the relationships between procrastination, self-regulation, and wellbeing. This study aims to investigate the moderation effect of self-regulation on the link between procrastination and life satisfaction among China and the United Kingdom.

## Literature Review

### Procrastination

Procrastination has been defined in a variety of ways, including “putting off acting on one’s intentions” (Lay and Silverman, 1996, p. 61). Schraw et al. (2007) defined procrastination as “intentionally deferring or delaying work that must be completed” (p. 13). However, Steel (2007) suggest that “to procrastinate is to voluntarily delay an intended course of action despite expecting to be worse off for the delay” (p. 7). Meanwhile, according to Lay and Silverman (1996), procrastination often appears in academic contexts, while students may procrastinate on assignments or examinations. Milgram et al. (1994) pointed out that academic procrastination is one specific form of procrastination, which was common among students (Senécal et al., 1995; Habelrih and Hicks, 2015).

According to Solomon and Rothblum (1984) and Senécal et al. (1995), procrastination is not simply about study skills or time management, but has behavioral, cognitive, and affective elements. The mechanism behind procrastination seems to be complicated. It is important to note that academic procrastination, as a widely studied specific type of procrastination in student groups, is different from general procrastination. Schraw et al. (2007) conducted a grounded theory study built a paradigm model of academic procrastination based on students’ reports. The paradigm model provides a framework for understanding academic procrastination and indicates that procrastination can be either positive or negative for students. Organization skill, as one of the self-characteristics, was one antecedent for procrastination in this model (Schraw et al., 2007). Flow, a situation when one completely devotes attention to a task (Czikszentmihalyi, 1990), was also highlighted in their study. Thus, procrastination has always been regarded as the failure of self-regulation (Sirois and Pychyl, 2013; Steel and Ferrari, 2013). Factors, such as attention control or self-regulation, appears to play an important role in procrastination, which has been proved in recent empirical studies (e.g., Abdi Zarrin et al., 2020; Hong et al., 2021).

Further, procrastination was reported to have limited impact on the students’ quality of work and either positive or negative impact on their life quality (Schraw et al., 2007). There are different types of procrastination. It could be either functional or dysfunctional (Ferrari, 1994), while we often tend to consider its dysfunctional side more. It could also be understood as active and passive procrastination (Choi and Moran, 2009; Habelrih and Hicks, 2015), as some individuals procrastinate because of their own choice of priorities. Self-regulated procrastination, as an approach to prioritizing tasks, appears to be different from dysfunctional or impulsive procrastination behaviors. Thus, it seems reasonable that not all procrastination behaviors are harmful to life and work as found in Schraw et al. (2007). The consequences of procrastination could be different across individuals, and it seems important to consider a wide range of factors, including self-regulation.

### Procrastination and Wellbeing

Empirical studies have proved the relationship between procrastination and wellbeing (e.g., Habelrih and Hicks, 2015; Grunschel et al., 2016; Meier et al., 2016; Duru and Balkis, 2017). The concept of wellbeing in those studies covers subjective wellbeing (including life satisfaction and positive and negative affects; Diener et al., 2002), psychological wellbeing (e.g., stress and anxiety), and physical health. Procrastination was found to predict subjective wellbeing especially among university students (Balkis, 2013; Grunschel et al., 2016; Duru and Balkis, 2017). For example, among 290 undergraduates, Balkis (2013) reveals that academic procrastination negatively predicts academic life satisfaction (*β* = −0.21, *p* < 0.001). Similarly, Duru and Balkis (2017) report that procrastination predicts subjective wellbeing (combing life satisfaction and positive and negative affects). Stress, as one factor of psychological wellbeing, was identified as a potential consequence of procrastination (Tice and Baumeister, 1997; Sirois and Tosti, 2012; Sirois and Kitner, 2015; Meier et al., 2016). Anxiety has also been proved to be associated with procrastination for many years (Solomon and Rothblum, 1984; Haycock et al., 1998; Fritzsche et al., 2003; Gagnon et al., 2016). Further, physical wellbeing (illness) was also found to be predicted by procrastination and mediated by stress (Sirois et al., 2003).

### Self-Regulation and Wellbeing

Self-regulation is always believed to be one of the reasons for health and happiness (Carver and Scheier, 1999; De Ridder and Gillebaart, 2017). The link between self-regulation (especially trait self-control) and wellbeing has been empirically proved by recent studies (e.g., Cheung et al., 2014; Hofmann et al., 2014). Trait self-control, as one dispositional component of self-regulation (Diehl et al., 2006), has been found to predict wellbeing among young adults and students (Cheung et al., 2014; Hofmann et al., 2014; Ronen et al., 2016). Hofmann et al. (2014) report that trait self-control significantly predicts life satisfaction mediated through affects. Among adolescents, Ronen et al. (2016) reveal that high self-control predicts higher subjective wellbeing moderated by social support. According to Cheung et al. (2014) trait self-control predicts happiness mediated through regulatory focus on goal pursuit. Therefore, the perceived impact of self-regulation on wellbeing seems clear, but the mechanism appears to be complex (mediation and moderation relationships).

### The Interaction of Self-Regulation and Procrastination on Wellbeing

Several studies show that procrastination mediates the relationship between self-regulation and wellbeing (Balkis and Duru, 2016; Grunschel et al., 2016). In other words, individuals feel unhappy probably because of their procrastination raised by poor self-regulation. This indicates the importance of considering self-regulation when investigating the consequences of procrastination. As personal organization skill (i.e., self-regulation) was argued as one of the antecedents for procrastination in Schraw et al. (2007), it appears that individuals with different levels of self-regulation can have different degrees of procrastination. Since both poor self-regulation and procrastination are proved to be predictors of low wellbeing, it thus seems interesting to explore whether there is an interaction effect of self-regulation and procrastination on wellbeing. It remains unknown whether the relationship between procrastination and wellbeing exists among individuals with high and low self-regulation. There could be a hypothesis that self-regulation moderates the relationship between procrastination and wellbeing.

### Culture and Procrastination

Cultural difference in procrastination has been investigated for many years. Ferrari et al. (2005) found that adults from the United Kingdom reported higher levels of arousal and avoidant procrastination than those from the United States and Australia. However, in another cross-cultural study, Ferrari et al. (2007) did not find significant difference on procrastination between participants from Europe, United States, and South America. A limited number of studies compared Eastern and Western people’s procrastination. Klassen et al. (2009) reveal that Singaporean adolescents perceived higher levels of procrastination than Canadian peers. However, they did not identify significant difference in terms of the links between procrastination and motivation variables (e.g., self-esteem and test anxiety). Similarly, Klassen et al. (2010) report that more Singaporean university students perceived themselves as negative procrastinators than Canadian students, but no difference for the link between procrastination and motivation variables. However, few studies (e.g., Klassen et al., 2009) have compared procrastination and its influences on wellbeing between East Asian and Western samples. It thus seems interesting to investigate whether cultural difference exists for the relationship between procrastination and wellbeing.

### Aim and Research Questions

According to the above discussion, it seems clear that either procrastination or self-regulation can be associated with people’s wellbeing, respectively. However, to our knowledge, no study explored the interaction effect of procrastination and self-regulation on wellbeing. It remains unknown whether procrastination always predicts lower wellbeing among individuals with difference levels of self-regulation. Furthermore, no study has compared this relationship between Eastern and Western groups. Thus, this study aims to investigate the following questions:

RQ1: What is the relationship between self-regulation, procrastination, and life satisfaction?

RQ2: To what extend does self-regulation moderate/affect the relationship between procrastination and life satisfaction?

RQ3: Are there any cultural differences across the two countries (China and United Kingdom) in terms of questions 1 and 2?

## Materials and Methods

### Participants and Procedure

Participants were 740 undergraduate students (265 British and 475 Chinese) recruited from two universities in south China and North England. The average age was 19.8 (*SD* = 1.11) in China and 20.3 (*SD* = 3.40) years in the United Kingdom. There were 209 female and 266 male Chinese students, and 219 females and 46 male British students. The Chinese data were obtained from a previous study (Yang et al., 2019) to compare with the British data. The participants were from different majors, including biology, business, computer science, English, education, management, and psychology. Questionnaires were distributed on campus of the two universities. For practical reasons, paper-based questionnaires printed in folded A3 papers were used in China. The Chinese participants were recruited during May and June in 2017. In the United Kingdom, both paper questionnaires and online survey in Qualtrics were distributed by the researcher during January to March in 2018. The participants read the consent information at the beginning of the questionnaires before deciding whether to the take the survey. They have the right to withdraw from this study before or during the study. All the answers are anonymous, and none of the participants can be tracked or identified in names.

### Measures

The survey collected the participants’ gender, age, and nationality. Three Likert scales were used to measure procrastination, self-regulation, and life satisfaction, respectively. The modified Irrational Procrastination Scale (IPS) and the Self-Regulation Scale (SRS) were translated into Chinese through a back-translation process and validated in Yang (2018). The validated Chinese version of the Satisfaction with Life Scale (SWLS) was adopted from Bai et al. (2011).

The modified 8-item IPS (Steel, 2010) was used to assess procrastination. It was a 5-point Likert scale answered from “Not at all true of me” to “Very true of me.” An example item is “At the end of the day, I know I could have spent my time better.”

The 10-item SRS (Diehl et al., 2006) was used to measure self-regulation. It was a 4-point Likert scale rated from “Not at all true” to “Completely true.” One of the items is “After an interruption, I do not have any problem resuming my concentrated style of working.”

The 5-item SWLS (Diener et al., 1985) was used to test life satisfaction. It was rated as a 7-point Likert scale from “Strongly disagree” to “Strongly agree.” A sample item is “If I could live my life over, I would change almost nothing.”

### Data Analysis

Descriptive statistics, correlation, and moderation analysis were conducted in SPSS version 24 and AMOS version 24. Multiple regression was used to analyze the moderation effect of self-regulation. Since there were only 46 males in the UK sample, considering gender difference was not the aim of the current study, gender difference was not included in data analysis. The Fisher’s transformation was applied to compare the correlation coefficients between the two countries. The interaction variable in the regression model was calculated through multiplying the standardized values (Z scores) of the IPS and SRS total scores. The process v3.4 (Hayes, 2017) program for SPSS was used to calculate the conditional effects of procrastination on life satisfaction at different values of self-regulation. Multi-group path analysis using structural equation modeling was conducted in AMOS. This study adopted the model fit indices, including *χ*^2^, χ^2^/*df* (rate of chi-square value and degree of freedom), comparative fit index (CFI), and root mean square error of approximation (RMSEA).

## Results

### Descriptive Statistics and Correlation Analysis

Table 1 shows the descriptive statistics of the three scales for procrastination, self-regulation, and life satisfaction in China and the United Kingdom. In both countries, the scales showed sufficient observed ranges and were normally distributed. The reliabilities of the scales were acceptable in both groups. The medians of corrected item-total correlations were all above 0.30.

**Table 1:** Descriptive scale statistics for the SRS in China and the United Kingdom.

Country	Scale	Range		Mean	SD	Skewness	Kurtosis	*α*	*MR*
		Potential	Observed						
China (*N* = 475)	IPS	8–40	11–40	25.14	4.74	−0.02	0.32	0.66	0.38
	SRS	10–40	13–40	26.16	3.71	0.06	1.12	0.69	0.37
	SWLS	5–35	5–35	18.35	5.37	0.17	−0.03	0.78	0.58
United Kingdom (*N* = 265)	IPS	8–40	9–40	26.22	6.72	−0.08	−0.58	0.89	0.67
	SRS	10–40	11–39	25.86	4.53	−0.22	0.29	0.79	0.47
	SWLS	5–35	6–35	23.23	6.58	−0.41	−0.47	0.87	0.70

*MR = median of corrected item-total correlations*.

Table 2 shows the Pearson product–moment correlation analysis for procrastination, self-regulation, and life satisfaction. In both countries, the three variables were significantly correlated with each other. Procrastination was negatively correlated with self-regulation (China: *r* = −0.39, *p* < 0.01, *r^2^* = 0.15; UK: *r* = −0.42, *p* < 0.01, *r^2^* = 0.18) and life satisfaction (China: *r* = −0.16, *p* < 0.01, *r^2^* = 0.03; UK: *r* = −0.32, *p* < 0.01, *r^2^* = 0.10) with small to medium effect sizes. Self-regulation and life satisfaction was positively correlated (China: *r* = 0.26, *p* < 0.01, *r^2^* = 0.07; UK: *r* = 0.40, *p* < 0.01, *r^2^* = 0.16) with small to medium effect sizes. In order to compare the correlations between the two countries, the correlation coefficient values were transformed into Z scores (Fisher’s transformation). Life satisfaction had significantly stronger correlations with and procrastination (*z* = −2.21, *p* < 0.05) and self-regulation (*z* = −2.04, *p* < 0.05) among the British students. Furthermore, no significant correlation was found between age and the three variables measured by the IPS, SRS, and SWLS in both countries. For the Chinese group, gender difference was only found for self-regulation. Males (*M* = 26.55, *SD* = 3.56) reported significant higher levels of self-regulation than females (*M* = 25.67, *SD* = 3.84) and *t*(473) = − 2.58, *p* < 0.05. For the British group, no significant gender difference was found for procrastination *t*(263) = − 1.74, *p* > 0.05; self-regulation *t* (263) = − 1.31, *p* > 0.05; and life satisfaction *t*(58.07) = 0.59, *p* > 0.05.

**Table 2:** Pearson product–moment correlations.

S. No.		China (*N* = 475)			United Kingdom (*N* = 265)		
		1	2	3	1	2	3
1	Procrastination	–			–		
2	Self-regulation	−0.39^**^	–		−0.42^**^	–	
3	Life satisfaction	−0.16^**^	0.26^**^	–	−0.32^**^	0.40^**^	–
4	Age	−0.04	0.02	−0.06	−0.08	0.00	−0.03
5	Gender	−0.07	0.12^*^	0.01	0.11	0.08	−0.04

* *p < 0.05 (two-tailed)*;

** *p < 0.01 (two-tailed)*.

*Significantly different correlation coefficients between groups are presented in bold*.

### Moderation Analysis

**Figure 1:**
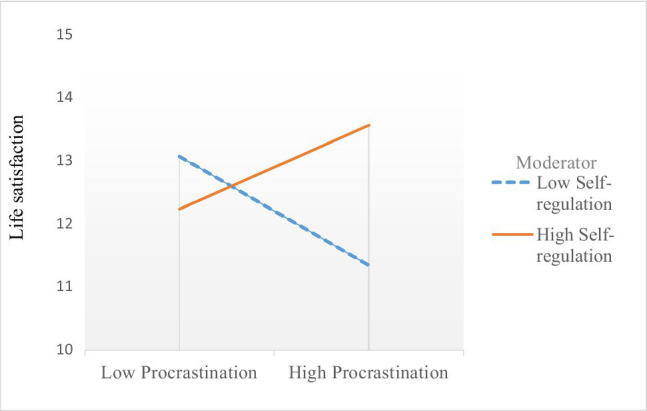
Simple slope figure for the moderation effect in the Chinse group (*N* = 475).

To test the moderation effect of self-regulation on the relationship between procrastination and life satisfaction, multiple regression analysis was conducted for the two groups separately. The interaction variable was calculated through multiplying the standardized values (Z scores) of the SRS and IPS. As given in Tables 3, a significant moderation effect was found only among the Chinese students (*β*= 0.13, *p* < 0.01). Self-regulation and procrastination had no interactive effect on life satisfaction among the British participants (*p* > 0.05). Figure 1 shows the moderation effect among the Chinese group.

**Table 3:** Regression coefficients for life satisfaction in China and the United Kingdom.

	China			United Kingdom		
	*B*	*β*	*p*	*B*	*β*	*p*
Gender	−0.32	−0.03	0.501	−0.96	−0.06	0.334
procrastination	−0.10	−0.09	0.067	−0.17	−0.18	0.005
Self-regulation	0.35	0.24	0.000	0.49	0.34	0.000
Interaction	0.76	0.13	0.004	−0.30	−0.07	0.242
*R*^2^		0.09			0.19	

*Interaction = Z score (procrastination) × Z score (self-regulation)*.

Structural equation modeling was applied to test the fitness of the moderation model in the two countries. In multi-group path analysis across the British and Chinese samples, the overall model fit was good, *χ*^2^ = 15.89, *df* = 4, *χ*^2^*/df* = 3.97, *CFI* = 0.95, *RMSEA* = 0.063, *NFI* = 0.94, *IFI* = 0.95, and *TLI* = 0.85 (*GFI* was not reported in AMOS because means and intercepts were estimated). All the paths were first constrained as equal. When the path from interaction to life satisfaction was freed (the other paths constrained), no significant chi-square change was identified, *Δχ*^2^ = 4.158, *df* = 2, *p* = 0.125. It shows that the interaction effect path was not different across the two countries. Self-regulation only moderated the effect of procrastination on life satisfaction among the Chinese sample. Self-regulation significantly predicts life satisfaction in both groups. When the path from self-regulation to life satisfaction was freed only, there is a significant chi-square change, *Δχ*^2^ = 9.39, *df* = 2, *p* = 0.009. There was also a significant chi-square change when the path from procrastination to life satisfaction was freed only, *Δχ*^2^ = 9.03, *df* = 2, *p* = 0.011. Thus, these two paths were not equal between the two groups, and no further comparison is needed.

**Figure 2:**
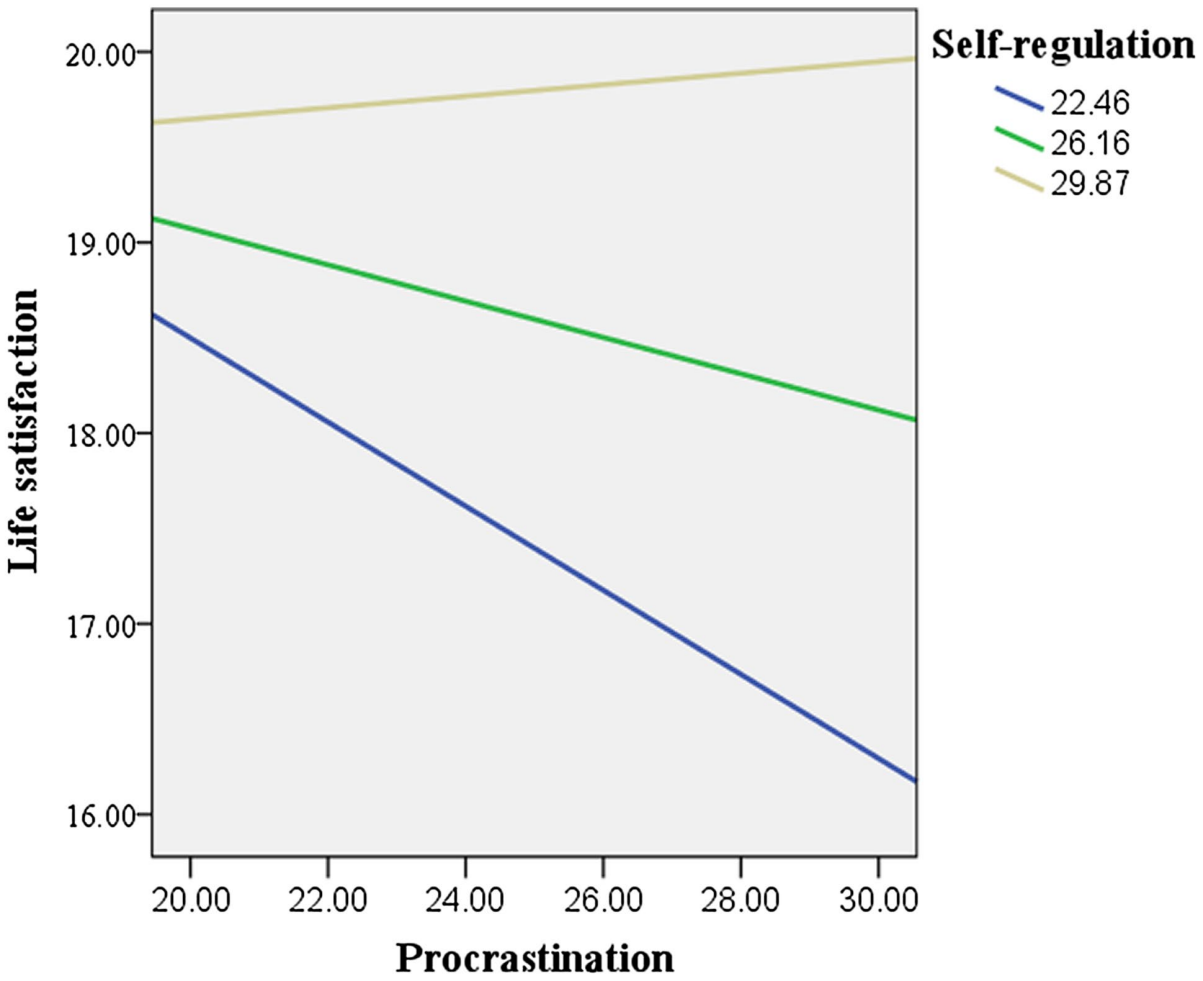
Moderation effect of self-regulation in China.

**Figure 3:**
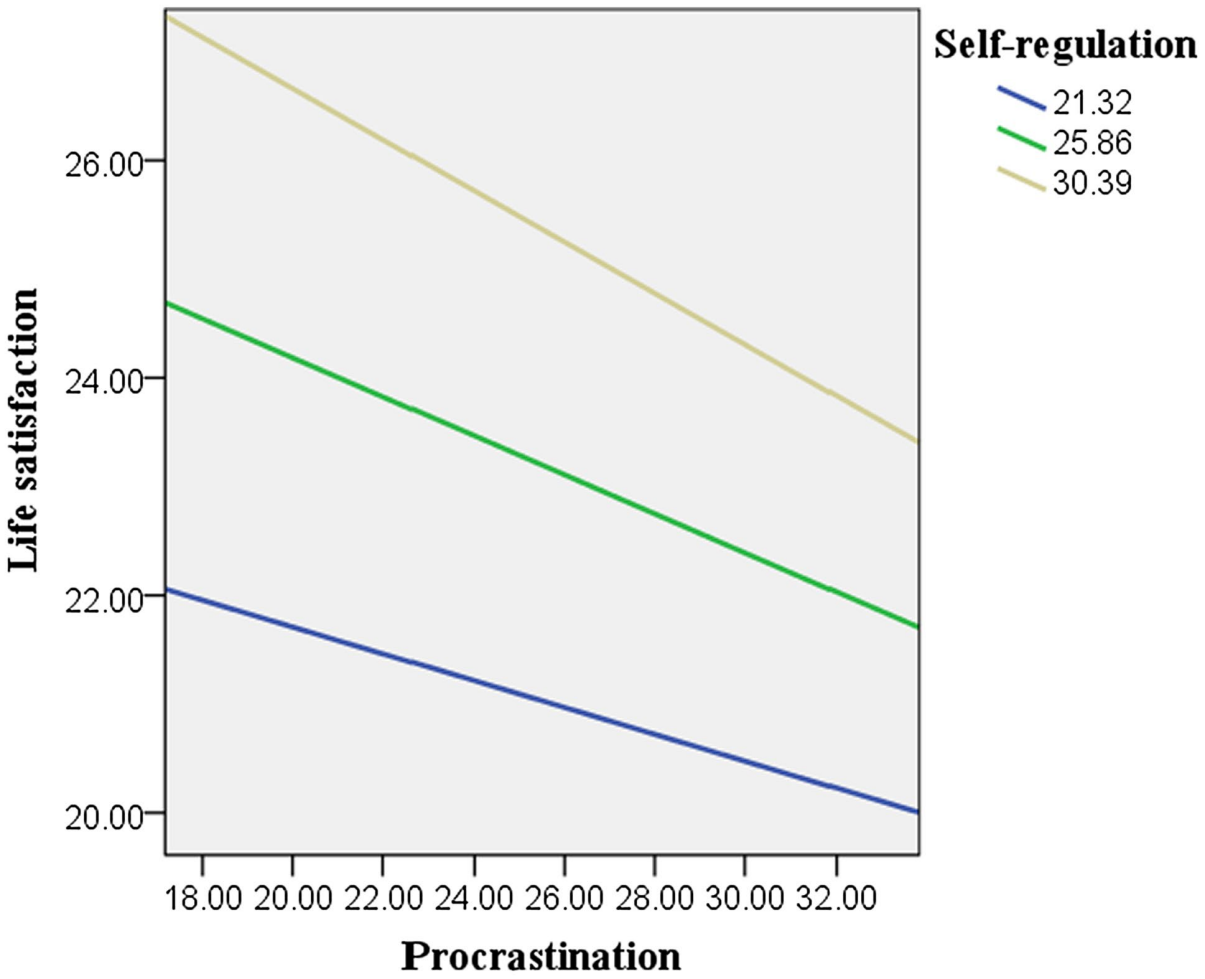
Moderation effect of self-regulation in the United Kingdom.

The process v3.4 (Hayes, 2017) in SPSS was used to calculate the conditional effects of procrastination on life satisfaction at different levels of self-regulation. As the moderation effect was significant only among the Chinese students, the conditional effects were only calculated for the Chinese data set. As given in Table 4, at lower levels (mean score minus one standard deviation) of self-regulation, procrastination significantly and negatively predicted life satisfaction (*γ* = −0.22, *t* = −3.03, *p* < 0.01). However, at higher levels of self-regulation, higher procrastination did not predict lower life satisfaction (*γ* = 0.03, *t* = 0.45, *p* > 0.05). This is also shown in Figures 2, 3, in which the moderation effect was significant in China but not in the United Kingdom.

**Table 4:** Conditional effects of procrastination on life satisfaction at different values of self-regulation in China.

Self-regulation		Effect	SE	*t*	*p*	LLCI	ULCI
*Mean − 1SD*	22.46	0.22	0.07	3.03	0.00	−0.36	−0.08
*Mean*	26.16	−0.10	0.05	−1.75	0.08	−0.20	0.01
*Mean + 1SD*	29.87	0.03	0.07	0.45	0.65	−0.10	0.16

### Mean Difference Comparisons for Procrastination, Self-Regulation, and Life Satisfaction

Independent samples’ *t* tests were used to compare the mean differences across China and the United Kingdom, as given in Table 5. The British participants reported significant higher levels of procrastination (*F* = 50.09, *p* < 0.001; *t*[423.39] = −2.33, *p* < 0.05, *d* = 0.20) and higher life satisfaction (*F* = 17.95, *p* < 0.001; *t*[460.81] = −10.30, *p* < 0.001, *d* = 0.84) than the Chinese peers. No significant difference was found for self-perceived self-regulation across the British and Chinese students.

**Table 5:** Mean differences for procrastination, self-regulation, and life satisfaction.

	China		United Kingdom		*F*	*t(df)*	*d*
	*N* = 475		*N* = 265				
	*M*	*SD*	*M*	*SD*			
Procrastination	25.14	4.74	26.22	6.72	50.09^***^	−2.33(423.39)^*^	0.20
Self-regulation	26.16	3.71	25.86	4.53	14.03^***^	0.941(461.84)	−0.08
Life satisfaction	18.35	5.37	23.23	6.58	17.95^***^	−10.30(460.81)^***^	0.84

* *p < 0.05 (two-tailed)*;

*** *p < 0.001 (two-tailed)*.

## Discussion

In summary, this study reveals that self-regulation moderated the relationship between procrastination and life satisfaction in China but not in the United Kingdom. Procrastination did not predict lower life satisfaction among the Chinese students who reported higher levels of self-regulation. The correlations between procrastination, self-regulation, and life satisfaction were significant in both countries, but stronger among the British students. Higher procrastination and lower self-regulation predicted lower life satisfaction in both countries. Procrastination and self-regulation were negatively associated. However, since all those results are based on self-report scales, no causal effect could be concluded. Furthermore, the British students reported significantly higher life satisfaction and procrastination than the Chinese students.

Previous study reveals that Singaporean students reported higher levels of procrastination than Canadian students (Klassen et al., 2009). In contrast, the present study found that the British students reported higher levels of procrastination than the Chinese students. It seems that the cultural difference in procrastination remains unclear in different contexts or countries. More cross-cultural studies are needed to investigate the cultural difference for the levels of procrastination. Klassen et al. (2009) found no significant difference between Singaporean and Canadian students for the relationships between procrastination and motivational variables (including self-efficacy for self-regulation). However, the current study found that self-regulation only moderated the relationship between procrastination and wellbeing among the Chinese students. It is necessary to further investigate whether cultural difference exist for the relationships between procrastination and self-regulation or other motivational variables.

In line with previous studies (Balkis, 2013; Grunschel et al., 2016; Duru and Balkis, 2017), this study confirms that procrastination is potentially linked with lower wellbeing (life satisfaction). Same as existing empirical evidence (Cheung et al., 2014; Hofmann et al., 2014; Ronen et al., 2016), this study identified that better self-regulation contributes to higher life satisfaction. Previous studies found the mediation role of procrastination in the link between self-regulation and wellbeing (Balkis and Duru, 2016; Grunschel et al., 2016). Similarly, this study finds that self-regulation moderated/affected the relationship between procrastination and life satisfaction in China. Besides the interaction between procrastination and self-regulation as expected, cultural difference appears to be an interesting factor for that moderation effect. The consequences of procrastination might not be consistent among people from different cultural backgrounds. This might explain why procrastination is not always harmful to life quality as reported in Schraw et al. (2007) theory of academic procrastination. Besides the three main antecedents of academic procrastination (self, teacher, and task) in their framework, cultural background can be another possible predictor for academic procrastination. In different cultures, the effects of procrastination might be different which can also be affected by other factors, for example, different self-regulation. The Chinese students with better perceived self-regulation were not affected by procrastination. It seems that those with higher self-perceived self-regulation were capable to handle their tasks well or more confident in finishing their tasks so that procrastination could not affect their lives. Such situation, however, might or might not be the same in other cultures, as in the British sample in this study. It is probably because of the different educational experience in China and the United Kingdom: Chinese students seem to be more adaptive to dealing with deadlines and academic stress (Yang, 2018). However, the reasons behind this cultural difference remain unclear only with the quantitative data in the current study. It thus seems necessary for further studies to explore why self-regulation only moderate the relationship between procrastination and life satisfaction in China, using qualitative designs.

There are some limitations for the present study. First, procrastination, self-regulation, and life satisfaction were measured by the three self-report scales. The potential bias of self-reported answers needs to be noted for this type of study because the participants might give socially desirable answers. Thus, the subjective measures in this study might limit the implications of the findings. Second, it is necessary to acknowledge that the current study did not include other factors of psychological wellbeing, such as stress or emotion. Future studies might need to focus on the potential mediation or moderation effect of the other wellbeing variables. Another limitation might be the purely quantitative design where only self-perceived scores for questionnaires were analyzed. Future studies might use mix-methods design to explore the effects of procrastination, comb questionnaire scores, interview narrative answers, and learn journals. Qualitative data might help to explain the reasons for the associations identified in quantitative data. The cross-sectional design is one limitation of this study. Therefore, in order to explore the relationships between procrastination and wellbeing, longitudinal design can be one of the potential directions in the future. It seems necessary to see whether procrastination or poor self-regulation consistently predicts lower wellbeing in a longer period (e.g., 3 months or a year).

## Data Availability Statement

The original contributions presented in the study are included in the article/supplementary material, further inquiries can be directed to the corresponding author.

## Ethics Statement

The studies involving human participants were reviewed and approved by Ethics committee of the Department of Education at the University of York. Written informed consent for participation has been obtained.

## Author Contributions

The first author designed the study, conducted data collection and data analysis, and wrote the manuscript.

### Conflict of Interest

The author declares that the research was conducted in the absence of any commercial or financial relationships that could be construed as a potential conflict of interest.
